# Effects of Whole-Body Electromyostimulation on Low Back Pain in People with Chronic Unspecific Dorsal Pain: A Meta-Analysis of Individual Patient Data from Randomized Controlled WB-EMS Trials

**DOI:** 10.1155/2017/8480429

**Published:** 2017-10-18

**Authors:** Wolfgang Kemmler, Anja Weissenfels, Michael Bebenek, Michael Fröhlich, Heinz Kleinöder, Matthias Kohl, Simon von Stengel

**Affiliations:** ^1^Institute of Medical Physics, Friedrich-Alexander University of Erlangen-Nürnberg, Henkestrasse 91, 91052 Erlangen, Germany; ^2^Department of Sports Science, University of Kaiserslautern, Erwin-Schrödinger-Strasse, 67663 Kaiserslautern, Germany; ^3^German Sport University Cologne, Am Sportpark Müngersdorf 6, 50933 Cologne, Germany; ^4^Department of Medical and Life Sciences, University of Furtwangen, Neckarstrasse 6, 78056 Villingen-Schwenningen, Germany

## Abstract

In order to evaluate the favorable effect of whole-body electromyostimulation (WB-EMS) on low back pain (LBP), an aspect which is frequently claimed by commercial providers, we performed a meta-analysis of individual patient data. The analysis is based on five of our recently conducted randomized controlled WB-EMS trials with adults 60 years+, all of which applied similar WB-EMS protocols (1.5 sessions/week, bipolar current, 16–25 min/session, 85 Hz, 350 *μ*s, and 4–6 s impulse/4 s impulse-break) and used the same pain questionnaire. From these underlying trials, we included only subjects with frequent-chronic LBP in the present meta-analysis. Study endpoints were pain intensity and frequency at the lumbar spine. In summary, 23 participants of the underlying WB-EMS and 22 subjects of the control groups (CG) were pooled in a joint WB-EMS and CG. At baseline, no group differences with respect to LBP intensity and frequency were observed. Pain intensity improved significantly in the WB-EMS (*p* < .001) and was maintained (*p* = .997) in the CG. LBP frequency decreased significantly in the WB-EMS (*p* < .001) and improved nonsignificantly in the CG (*p* = .057). Group differences for both LBP parameters were significant (*p* ≤ .035). We concluded that WB-EMS appears to be an effective training tool for reducing LBP; however, RCTs should further address this issue with more specified study protocols.

## 1. Introduction

Low back pain (LBP) is one of the leading causes of chronic diseases worldwide [1, 2]. In Western Europe and North America, LBP was the most common source of disability-adjusted life-years in 2013, without any positive tendency [1, 2]. Severe and chronic LBP increases with advanced age [3] and usually results in functional disability and loss of independence; thus effective LBP management in older adults is crucial [4]. In about 80% of the patients, the causes for chronic LBP are nonspecific [5], or best practice therapies were challenging. Physical exercise is a recognized agent in the area of unspecific chronic LBP [6, 7], but the enthusiasm for exercise is not pronounced in older people with chronic LBP. Lack of time was reported as the main obstacle to exercise [8]; furthermore kinesiophobia, that is, the fear of pain due to movement, is very prevalent in this cohort [9]. Alternative training technologies that overcome the prevalent limitations of conventional exercise may be promising options for people with chronic unspecific LBP. Whole-body electromyostimulation (WB-EMS), a time-efficient, safe, and joint-friendly technology, may be such a choice [10, 11]. However, although many commercial providers promote WB-EMS as an effective therapy for LBP, the scientific evidence for this assertion is rather vague. In fact, only one nonpublished university report focuses on this issue [12]. The aim of this study is to provide evidence for the effect of WB-EMS on chronic, unspecific LBP in older people. For this project, we conducted a meta-analysis of individual patient data of five of our recent WB-EMS studies [10, 11, 13–15] with older people, with special focus on participants with frequent-chronic, unspecific pain at the lumbar spine (LS).

Our primary hypothesis was that WB-EMS significantly decreases pain intensity at the LS in older people with chronic, unspecific LBP. Our secondary hypothesis was that WB-EMS significantly decreases pain frequency at the LS in older people with chronic, unspecific LBP.

## 2. Methods

The aim of the study was to compare the effects of WB electromyostimulation versus nontraining control on LBP in older people. To adequately address our hypothesis we conducted an analysis of individual patient data derived from 5 randomized controlled WB-EMS trials (RCT) with parallel group designs (WB-EMS versus control) [10, 11, 13–15] carried out between 2010 and 2017. All the trials were planned and conducted by the Institute of Medical Physics (IMP), University of Erlangen (FAU), Germany, complied with the Declaration of Helsinki “Ethical Principles for Medical Research Involving Human Subjects,” and were approved by the ethics committee of the FAU (numbers 67_15b, 301-13B, 4184, 3876, and 3777).

### 2.1. Participants

For details of the recruitment processes of the trials, the reader is kindly referred to the corresponding studies [10, 11, 13–15]. For the present analysis, we initially selected studies that (1) applied a similar WB-EMS protocol for more than 14 weeks; (2) included only people without previous WB-EMS experience; (3) used the same pain questionnaire; (4) focused on cohorts predominately 60 years and older; (5) included people living independent in the community; and (6) applied a randomized controlled trial (RCT) approach with parallel group designs (WB-EMS versus control).

Five of our recent WB-EMS trials [10, 11, 13–15] with altogether 310 male (*n* = 129) and female (*n* = 181) participants satisfied these criteria.

For a participant base, we also retrospectively checked eligibility of the subjects applying the inclusion criteria: (1) unspecific low back pain, (2) frequent to chronic pain at the lumbar spine (LS), (3) at least moderate pain intensity at the LS, (4) patients being 60 years and older, and (5) complete data sets for the primary and secondary endpoints discussed below. In summary, 45 study participants (WB-EMS: *n* = 23; control: *n* = 22) who fully met our eligibility criteria were finally identified and included in the analysis (Table 1).

### 2.2. Procedures

#### 2.2.1. Primary Study Endpoint

- This includes intensity of back pain at the lumbar spine.

#### 2.2.2. Secondary Study Endpoint

- This includes frequency of back pain at the lumbar spine.

### 2.3. Measurements

In general, in each of the studies participants were tested at baseline and follow-up by the same researcher at the same time of the day (±1 hour). All the assessments were determined in a (semi)blinded mode; testing staff and outcome assessors were unaware of the participant status (i.e., WB-EMS or control).

#### 2.3.1. Study Outcome

Pain intensity and pain frequency were determined by a questionnaire validated in two randomized controlled studies with older cohorts [18, 19]. In detail, this questionnaire asked for frequency and maximum pain intensity at the spine (cervical spine, thoracic spine, and lumbar spine) and main joints during the last 4 weeks using a 0–7 scale. “0” represented “no pain”; “7” indicated “chronic pain” (pain frequency) or very severe, unbearable pain (pain intensity). Participants were included in the study when reporting ≥“5” for pain frequency and ≥“4” for pain intensity at the LS. “5” indicated “frequent to very frequent” pain sensation (or severe pain intensity) and “4” “moderate” pain intensity.

Nonspecificity of low back pain was monitored by the evaluation of baseline and follow-up data derived from questionnaires that addressed diseases, injuries, medications and lifestyle, and, where appropriate, medical documents. Two researchers (WK, SvS) independently checked the data. In doubtful cases (i.e., osteoporosis without diagnosed vertebral fractures), subjects were not included in the present study.

#### 2.3.2. Anthropometry

Body height and body mass were measured by calibrated devices. Body Mass Index was calculated by weight (kg)/height (m^2^).

#### 2.3.3. Confounding Factors

The same standardized questionnaire was applied in all the studies to determine confounding factors that might affect the projected outcome parameters. Apart from lifestyle, diseases and medications, demographic data and general health risk factors (alcohol, smoking) were also assessed at baseline and follow-up. Baseline status and changes of physical activity and exercise were determined by specific questionnaires [16] and personal interviews.

### 2.4. Interventions

Primary study endpoints of the five small- to medium-sized RCTs (*n* = 28–101) included focused on muscle mass and strength/functional abilities. One study also addressed Bone Mineral Density [13, 20]. Study duration varied between 14 weeks [14, 15] and 12 months [13].

#### 2.4.1. Whole-Body Electromyostimulation (WB-EMS)

**Figure 1:**
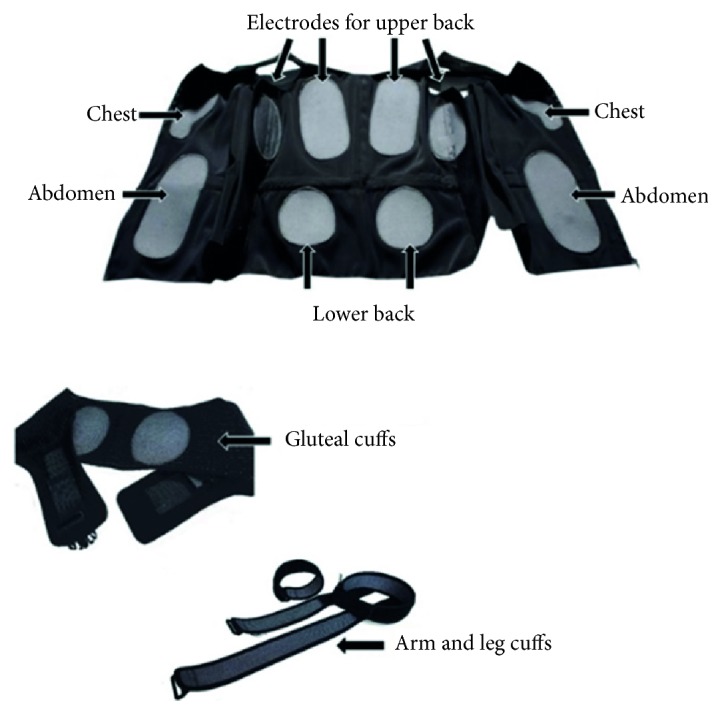
WB-EMS electrodes (grey area) of the WB-EMS equipment used in the underlying trials.

**Figure 2:**
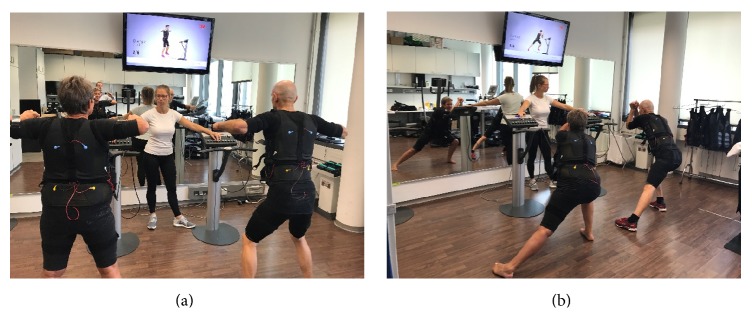
WB-EMS training setting with one instructor and two applicants [17].

All the studies scheduled comparable WB-EMS protocols. We consistently used the same WB-EMS devices (miha bodytec, type I, Gersthofen, Germany) and stimulated the same main muscle groups (Figure 1). We applied bipolar electric current, selected an impulse frequency of 85 Hz, an impulse width of 350 *μ*s, and used an interval approach with 4–6 sec of stimulation and 4 sec of rest. In two studies, however, we prescribed an additional continuous WB-EMS application with 7 Hz for 10 min [14] or 15 min [15]. In addition, consistently, slight, low intensity/low amplitude dynamic exercises were performed (Figures 2(a) and 2(b)) during the 4–6 s stimulation phase of the studies. In each of the studies emphasis was placed on exercise that should not affect muscle parameters per se. Training frequency varied between 1 [11] and 1.5 sessions per week [10, 13–15] and the duration of the sessions also ranged from 16 to 25 min. The intensity of the stimulation was consistently regulated using the Borg CR 10 [21] “rate of perceived exertion” (RPE) scale. For each of the muscle groups stimulated, participants were encouraged to exercise at an RPE of “5-6” (i.e., hard to hard+) [11] and “6-7” (i.e., “hard+ to very hard”) [10, 13–15] on the Borg CR10 scale.

All studies applied a consistently supervised video-guided setting with one instructor and two participants (Figures 2(a) and 2(b)). For more details, the reader is kindly referred to previous publications (e.g., [13]).

#### 2.4.2. Control

Apart from one study [15] that focused on participant blinding, all other studies implemented nontraining control groups that were asked to strictly maintain their habitual lifestyle during the study period. The “active” control group of the former study applied a slight movements/exercises program on whole-body vibration (WBV, 30 Hz) platforms 1.5 × 18 min/week with special regard to flexibility.

### 2.5. Statistical Analysis

Based on a recent meta-analysis [7] that compared the effects of conventional types of exercise on low back pain, we conservatively expected a standardized mean difference (SMD) between WB-EMS and control of 0.40 ± 0.45. Correspondingly we aimed to include 20 persons per group to validate a corresponding difference with *α* = .05 and *β* − 1 = 0.8.

All the participants of the WB-EMS and the control groups were correspondingly pooled in one WB-EMS versus one control group and compared without assigning weights to an underlying study or group of study participants.

After checking the baseline data given in Table 1 and study endpoints for normal distribution by QQ plots, the data were reported as mean value (MV) ± standard deviation (SD) and 95% confidence interval (CI). Within-group differences were calculated with paired *t*-tests; differences between WB-EMS and control were analyzed with the Welch *t*-test. Chi-Square tests were applied to detect difference in nominal scaled (baseline) data. All tests were 2-tailed, statistical significance was accepted at *p* < .05. Effect sizes (ES) were calculated using SMD (i.e., group difference/pooled SD). ES ≥ 0.5 were considered as moderate; ES ≥ 0.8 were considered as high. SPSS 22.0 (SPSS Inc., Chicago, IL) was used for all statistical procedures.

## 3. Results

### 3.1. General Results

The number of participants included from each study varied from four [14] to 13 [13]. Although the rate of eligible participants in WB-EMS versus CG differed between the underlying studies (e.g., [15], 4 : 8, versus [10], 6 : 3), all the studies provided participants for both groups. Baseline characteristics of the included subjects did not vary significantly between the five WB-EMS trials. One exception was the baseline training status of the participants, however. While four trials focused on physically untrained older people (≤1x resistance type exercise/w.), the first of our WB-EMS studies [14] included women with a number of years of experience in resistance exercise training.

Attendance of the WB-EMS classes averaged 92% (individual range 79–100%); all participants of the CGs and WB-EMS groups reported that they had maintained their normal lifestyle during the study phases.

Table 1 lists the baseline results of the pooled WB-EMS and CG. In conclusion, no significant differences were observed for baseline parameters and/or parameters that may affect our study results.

### 3.2. Main Outcome Parameters

Table 2 lists the baseline, follow-up, and corresponding changes and group differences for primary and secondary outcomes. About 40% of each of both groups reported suffering from frequent (“5”) or very frequent (“6”) LBP; 22% listed permanent low back pain during the last 4 weeks. Correspondingly maximum pain intensity at the LS was moderate in 20%, high in 45%, and very high in about 35% of the participants.

No significant group differences were observed at study start for pain intensity and frequency at the LS (*p* ≥ .563). Pain intensity at the LS decreased significantly in the WB-EMS group (*p* < .001) and was unchanged in the CG (*p* = .997). Differences between the groups were significant (*p* = .008); the effects size can be considered high. Thus, we confirmed our primary hypothesis that WB-EMS significantly decreases pain intensity at the LS in older people with frequent-chronic, unspecific LBP.

Pain frequency at the LS changed favorably in the WB-EMS group (*p* < .001) and showed a borderline nonsignificant improvement in the CG (*p* = .057). WB-EMS and CG differ significantly for this pain parameter (*p* = .035); effect size for this outcome was moderate to high. Correspondingly, we confirmed our secondary hypothesis that WB-EMS significantly decreases pain frequency at the LS in older people with at least frequent, unspecific LBP.

As mentioned in Table 2, WB-EMS and CG did not vary considerably for baseline LBP parameters. Further, changes in pain intensity and frequency did not vary relevantly between the WB-EMS groups of the five studies included in this analysis. In contrast, study-specific changes in the CG differ considerably for pain frequency (and to a lesser degree for pain intensity), with the most favorable changes in the active control group that conducted a slight WBV approach [15].

### 3.3. Potentially Confounding Parameters

No participant of the WB-EMS or CG reported changes of lifestyle including physical activity, exercise, diet, and medication including analgesic agents during the study phases of the underlying trials.

## 4. Discussion

In the present contribution, we provide a considerable body of evidence for the favorable effect of WB-EMS on low back pain in persons affected by such problems. We thus confirmed the unpublished university report of Boeckh-Behrens et al. [12] which determined the positive effects of a more time consuming (2 × 45 versus 2 × 20 min/w.) but otherwise comparable WB-EMS application (bipolar, 85 Hz, 350 *μ*s, 4 s impulse-2 s rest), on dorsal pain in a cohort of 49 adults with infrequent back pain. Without a nontraining control group, the authors reported a reduction of dorsal pain frequency in 89% of their WB-EMS applicants, which is higher than the 70% responder rate for both the intensity and frequency of LBP determined in the present study. The effect size of our finding (Table 2) can be considered moderate (LBP frequency) to high (LBP intensity). Comparing our results with conventional exercise in the area of LBP therapy, there is a more favorable effect on LBP than the average effect of strength/resistance exercise (SMD: 0.50) or coordination/stabilization exercise (SMD: 0.47) reported by a recent meta-analysis [7] that included 39 RCTs. However, two resistance [22, 23] and two stabilization [24, 25] exercise protocols were more effective (SMD: 1.58–2.27) for decreasing LBP than the present study. With respect to the feasibility, safety, and attractiveness of these studies for a cohort of chronic LBP, albeit with one exception that applied a single 20 min isolated back extensor strengthening session per week [23], all of the protocols were much more time consuming than the present protocol. Furthermore, all of the trials focused on predominately dynamic exercises with relevant loading [22–24] and/or moderate to full range of motion in lumber extension [22–25], a feature that might conflict with the kinesiophobia shown by many chronic LBP patients.

Summarizing the effectiveness of WB-EMS in the area of low back pain, we observed a moderate to high positive effect in people with unspecific, frequent to chronic LBP. This result was not necessarily to be expected. Reviewing the literature, RCTs applying transcutaneous electrical nerve stimulation (TENS), a locally applied version of electrostimulation dedicated to LBP, showed conflicting results (review in [26, 27]). In Germany TENS is not recommended for the therapy of LBP, whereas the German National LBP Guideline [28] contraindicates this technology for acute and chronic unspecific LBP due to the “passive” application. However, a recent RCT provided additional information about the efficacy of different electrical therapies that focus on chronic LBP [27]. This pilot study compared the effects of various electrical therapies including TENS, acupuncture-like TENS, high voltage TENS, inferential current (IFC), and stabilization exercises on pain intensity (all 5 × 20 (IFC), 60 min/w. (TENS) for 3 weeks). Briefly, all the therapies significantly reduce LBP; however the impacts of the electrical therapies listed above were significantly more effective compared with the conventional exercise group.

In our study, we applied a WB-EMS protocol very much like the most popular commercial WB-EMS application, that is, all main muscle groups, bipolar current, 85 Hz, 350 *μ*s, and rectangular 4–6 s impulse phase/4 s of rest with slight dynamic exercises during the impulse phase. Of importance, unlike the majority of electrical applications for LBP, we consistently focused on high (strain) intensity, scheduled by a rate of perceived exertion of “5-6” (i.e., hard to hard+) on the Borg CR10 scale [21] for each of the 8 electrode sites (Figure 1). This aspect demonstrates that supervision and close feedback between instructor and applicant are crucial in WB-EMS in order to adequately generate the prescribed strain intensity and to properly conduct the exercises/movements in this older cohort of predominately less sportive people with low body awareness. In parallel, close supervision was also reported to be a key aspect of successful LBP protocols [29, 30].

Apart from this close supervision of WB-EMS, other potential causes might generate pathways supplemental to the analgesic effect of conventional exercise. Firstly, favorable neuromodulation effects are suggested according to the “gate control theory” [31], which hypothesizes that transmission of pain is inhibited by the electrical stimulation of large, afferent nerve fibers. Further mechanisms of pain reduction of opioid-mediated analgesia were reported after intense, high frequency TENS application [32], a method similar to our strain protocol. Both pathways might explain a favorable acute and short-term effect of electrical stimulation on LBP; the corresponding long-term effect might be explained by the spinal muscular adaptions reported for WB-EMS [14, 33] generating an increased segmental stabilization of the spine.

### 4.1. Limitation of Following Research

In order to allow the reader to put the results of the present study in a better context, we would like to address some of its limitations and specific features. (1) This study can be regarded as a meta-analysis of individual patient data; thus the potential pitfalls of meta-analytic approaches should be borne in mind [34]. One of the most important issues for meta-analysis approaches may be heterogeneity or more precisely the threshold up to which a study and, in our case, a study participant can still be meaningfully included. In summary, with the exception of the intervention periods which vary from 14 to 52 weeks, we conclude that the included studies [10, 11, 13–15] were very consistent with respect to their WB-EMS interventions. With respect to the corresponding study cohorts, the variety of potentially confounding parameters might be higher. In actual fact, the cohorts vary from participants with “Sarcopenic Obesity” [10, 11] to people with Metabolic Syndrome [15]. As for the participants, we focused on people aged 60+ with unspecific, frequent-chronic low back pain of at least moderate intensity without further consideration of their health, fitness, or exercise status, which also varies considerably between the study cohorts of the underlying trials. However, although sample size was too low to conduct a dedicated analysis, the favorable effect of WB-EMS on LBP did not differ between the underlying RCTs, indicating that WB-EMS was effective in older cohorts with LBP largely independent of the health, fitness, and exercise status.

(2) Most crucially, none of the underlying trials focused on “unspecific chronic low back pain” as the primary endpoint. Correspondingly, we did not apply an LBP-specific assessment tool but used our recognized questionnaire that addresses pain frequency and intensity at the spine (i.e., cervical, thoracic, and lumbar spine) and main joints. Although we consistently used this questionnaire and checked the data carefully together with the participants, we have to admit that we do not clearly quantify pain frequency (*h*/*d*; *d*/*w*). Thus, our inclusion criteria of pain frequency ≥“5,” that is, “frequent” during the last 4 weeks, was somewhat vague. As a result, people with minor LBP problems might be included, leading to a potentially lower WB-EMS effect due to limited improvement prospects. From a methodological point of view, however, this restriction makes our finding more cautious and discrete and was thus of lower relevance. More importantly, however, the assessment of the nonspecificity of LBP was quite difficult even though we properly determined and monitored medical history including diseases and injuries in all of our trials. However, due to the retrospective character of this work, the two researchers who independently assessed the present participant data have only a limited opportunity to clear up doubtful cases (*n* = 9) together with the participant in question. Although all the doubtful cases were excluded, we cannot be completely sure that LBP was consistently unspecific in all of our subjects.

An important strength of our approach however is that all but one [14] of the underlying studies were “state-of-the-art” RCTs that included only physically inactive older adults, that is, a cohort which can be regarded as the key target group for carefully supervised WB-EMS applications. Also of relevance for older people, unlike spine-specific TENS applications or isolated back extensor strengthening [23], the applied WB-EMS protocols additionally improve body composition, strength, and physical functioning [10, 11, 13–15, 33]. Besides its analgesic effect on LBP, WB-EMS can be considered as a promising, time-effective, safe, and joint-friendly therapy option especially for multimorbid older adults. The participants' high acceptance of WB-EMS reflected by the low dropout and high adherence rates reported by all of our previous WB-EMS trials with older adults [10, 11, 13–15, 33] might underscore this estimation.

With respect to the transferability of our results, we assume that the only minor variation of LBP-changes among the cohorts and participants included might be legitimate to enlarge the scope of our finding to older people with frequent to chronic unspecific LBP regardless of their fitness and exercise status, although this has to be verified in detail.

## 5. Conclusion

In summary, we see our results more as a preliminary finding than definitive evidence justifying a conclusion that WB-EMS has a favorable effect in the treatment of chronic, unspecific LBP. More dedicated WB-EMS RCTs with sufficient statistical power that focus on a homogeneous cohort of people with definite chronic and unspecific LBP and which incorporate generally accepted pain questionnaires that specifically focus on the low back region should be conducted to finally conclude this issue. Until then, WB-EMS should be regarded as a promising but still not adequately verified therapy for addressing chronic unspecific low back pain in the elderly.

## Figures and Tables

**Table 1:** Baseline characteristics of the participants of the HIT and control groups.

Variable	WB-EMS *n* = 23	Control *n* = 22	Difference (*p*)
Gender [women/men]	12/11	14/8	.606

Age [years]	72.0 ± 7.1	72.5 ± 7.8	.429

Body height [cm]	166.3 ± 9.9	166.0 ± 8.4	.926
Body weight [kg]	71.7 ± 9.4	68.8 ± 10.4	.315
Physical activity [index]^a^	2.91 ± 1.08	3.22 ± 1.51	.463
Exercise volume [min/week]	41.0 ± 37.8	50.2 ± 35.2	.689
Number of diseases [*n*]	3.4 ± 1.7	3.0 ± 1.4	.331
Number of orthopedic diseases [*n*]	2.2 ± 0.8	2.0 ± 0.9	.601
Smoker [*n*]	8	8	.912

^a^Self-rated physical activity score (1, very low, to 7, very high) [16].

**Table 2:** Baseline, absolute changes, and statistical parameters of the primary and secondary outcomes in the HIT and control group. ^*∗*^*p* < .05; n.s.: nonsignificant.

	WB-EMS (*n* = 23) (MV ± SD)	Control (*n* = 22) (MV ± SD)	Difference MV (95%-CI)	*p*	SMD
*Pain intensity at the lumbar spine (LBP) *[index]^a^					
Baseline	5.13 ± 0.87	5.23 ± 0.87	—	.619	—
Postintervention	4.26 ± 0.92	5.23 ± 0.81	—	—	—
Difference	−0.87 ± 1.06^*∗*^	0.00 ± 1.02^n.s^	.87 (0.24 to 1.50)	.008	0.84

*Pain frequency at the lumbar spine (LBP) *[index]^b^					
Baseline	5.78 ± 0.77	5.86 ± 0.78	—	.563	—
Postintervention	4.87 ± 0.82	5.50 ± 0.86	—	—	—
Difference	−0.91 ± 0.85^*∗*^	−0.36 ± 0.85^n.s^	0.64 (0.04 to 1.06)	.035	0.65

^a^Index from 0 (no pain) to 7 (unbearable pain): WB-EMS. ^b^Index from 0 (no pain) to 7 (chronic pain): WB-EMS.
